# Clinical factors associated with 30-day heart failure readmission in patients with acute decompensated heart failure with preserved ejection fraction

**DOI:** 10.3389/fcvm.2026.1891685

**Published:** 2026-08-19

**Authors:** Kun Yin, Qiao Liu, Wanzhen Yin, Yangqiang Xu, Yongchao Zhang

**Affiliations:** 1 Department of Cardiology, Anyue People's Hospital, Ziyang, Sichuan, China; 2 Department of Laboratory Medicine, Anyue People's Hospital, Ziyang, Sichuan, China; 3 Outpatient Department, Anyue People's Hospital, Ziyang, Sichuan, China; 4 Department of Cardiology, The First Affiliated Hospital, Zhejiang University School of Medicine, Hangzhou, Zhejiang, China; 5 Cardiology Department, Chongqing Jiulongpo District People's Hospital, Chongqing, China

**Keywords:** heart failure with preserved ejection fraction, acute decompensated heart failure, 30-day readmission, risk factors, transitional care

## Abstract

**Background:**

In acute decompensated heart failure with preserved ejection fraction (ADHFpEF), residual congestion before discharge is closely related to early heart failure (HF) readmission. This study evaluated the clinical factors associated with 30-day HF readmission in patients with ADHFpEF.

**Methods:**

This single-center retrospective study included 312 adults discharged after hospitalization for ADHFpEF between January 2020 and December 2024. Clinical, laboratory, echocardiographic, medication, and follow-up data were obtained from the medical records. Factors associated with 30-day HF readmission were evaluated using univariable and multivariable logistic regression analyses. Medication adherence was assessed using the Chinese version of the Adherence to Refills and Medications Scale (ARMS-C). Sensitivity analyses addressed coronavirus disease 2019 (COVID-19)-related calendar periods and chronic obstructive pulmonary disease (COPD).

**Results:**

Thirty-day HF readmission occurred in 62 patients (19.9%). Adherence and sensitivity analyses were consistent with the primary findings.

**Conclusions:**

In patients with ADHFpEF, several clinical factors were associated with 30-day HF readmission. Identification of these factors may support early risk assessment and individualized post-discharge management.

## 1 Introduction

Heart failure with preserved ejection fraction (HFpEF) is a major clinical phenotype of heart failure (HF), and its disease burden, risk of rehospitalization, and use of healthcare resources continue to increase. Early events after acute decompensation are often concentrated in the first few weeks after discharge, and 30-day HF readmission remains an important indicator of pre-discharge risk status and the quality of transitional care (1). HFpEF is highly heterogeneous and is commonly accompanied by advanced age, obesity, atrial fibrillation, renal dysfunction, inflammatory activation, and elevated pulmonary circulatory pressures, making it difficult for any single clinical measure to fully explain the risk of short-term recurrent decompensation (2).

Unlike heart failure with reduced ejection fraction (HFrEF), patients with HFpEF may maintain a normal or near-normal left ventricular ejection fraction (LVEF), yet still have elevated left ventricular filling pressure, pulmonary venous congestion, and hemodynamic abnormalities at rest or during exercise. LVEF alone does not reflect volume load, filling pressure, or residual congestion and cannot adequately explain the short-term risk that persists despite symptomatic improvement (3). Current management of HFpEF emphasizes integrated assessment of the clinical phenotype, volume status, renal function, natriuretic peptide levels, and comorbidities, while pre-discharge residual congestion remains an identifiable and potentially modifiable driver of readmission (4).

Clinical symptoms, signs, and natriuretic peptide levels in HFpEF are readily influenced by age, obesity, renal function, atrial fibrillation, and inflammatory status and may therefore underestimate subclinical residual volume overload (5). N-terminal pro-B-type natriuretic peptide (NT-proBNP) provides important prognostic information, but its level is affected by renal function, body weight, and cardiac rhythm and cannot fully replace bedside assessment of volume status (6, 7).

Early identification of patients at increased risk of readmission may facilitate individualized discharge planning, closer follow-up, and optimization of transitional care (8, 9). Using data from patients hospitalized with ADHFpEF between January 2020 and December 2024, this study examined the clinical factors associated with 30-day HF readmission.

## 2 Materials and methods

### 2.1 Study population

This single-center retrospective study consecutively included patients hospitalized with ADHFpEF in the Department of Cardiology at Anyue People's Hospital between January 2020 and December 2024 who were subsequently discharged. After application of the inclusion and exclusion criteria, 312 patients were included in the primary analysis. The study protocol was reviewed and approved by the Medical Ethics Committee of Anyue People's Hospital (approval no. AY 20260523003). Because the study used previously collected clinical data and did not interfere with diagnostic or therapeutic procedures, the informed-consent process was conducted in accordance with the requirements of the Medical Ethics Committee of Anyue People's Hospital.

Acute decompensated heart failure (ADHF) was diagnosed on the basis of acute or subacute onset of dyspnea, fatigue, peripheral edema, pulmonary crackles, pulmonary congestion, or other clinical manifestations, together with elevated natriuretic peptide levels and imaging or echocardiographic evidence supporting cardiac structural or functional abnormalities (10). HFpEF was defined as LVEF ≥ 50%, HF-related symptoms and signs, and objective evidence such as left atrial enlargement, left ventricular hypertrophy, diastolic dysfunction, or elevated natriuretic peptide levels (11).

The inclusion criteria were as follows: (1) age ≥18 years; (2) ADHFpEF as the principal discharge diagnosis; (3) clinical stabilization after anti-HF treatment followed by discharge; and (4) availability of readmission and mortality information within 30 days after discharge.

The exclusion criteria were as follows: (1) LVEF <50%; (2) acute coronary syndrome, severe valvular heart disease, congenital heart disease, constrictive pericarditis, or hypertrophic obstructive cardiomyopathy; (3) receiving mechanical ventilation, having a tracheostomy, or being unable to cooperate with spontaneous breathing at the time of examination; (4) acute exacerbation of chronic obstructive pulmonary disease (COPD), active pneumonia or another acute respiratory infection, coronavirus disease 2019 (COVID-19) pneumonia within the preceding 90 days, persistent noncardiogenic pulmonary parenchymal abnormalities on chest imaging before discharge (including residual post-COVID-19 pulmonary changes), interstitial lung disease, pulmonary fibrosis, large pleural effusion, confirmed cirrhosis or moderate-to-large ascites, or another condition that could affect the assessment of clinical volume status; (5) end-stage kidney disease requiring maintenance dialysis, advanced malignancy, or expected survival <30 days; (6) missing key clinical data or 30-day outcome data; and (7) in-hospital death or failure to be discharged.

### 2.2 Clinical data collection

Baseline data were retrieved from the electronic medical record system, including age, sex, body mass index, smoking history, New York Heart Association (NYHA) functional class, length of stay, systolic blood pressure, diastolic blood pressure, heart rate, and medical history. Medical history included hypertension, diabetes mellitus, coronary artery disease, atrial fibrillation, chronic kidney disease, COPD, and previous stroke.

Laboratory measures were obtained from the most recent tests before discharge and included hemoglobin, serum creatinine, blood urea nitrogen, serum sodium, serum potassium, albumin, high-sensitivity C-reactive protein (hs-CRP), NT-proBNP, and estimated glomerular filtration rate (eGFR). Echocardiographic parameters included LVEF, left atrial volume index (LAVI), interventricular septal thickness, the ratio of early mitral inflow velocity to early diastolic mitral annular velocity (E/e′), and pulmonary artery systolic pressure (PASP). Discharge medications were also recorded, including loop diuretics, mineralocorticoid receptor antagonists (MRAs), β-blockers, angiotensin-converting enzyme inhibitors (ACEIs) or angiotensin II receptor blockers (ARBs), angiotensin receptor-neprilysin inhibitors (ARNIs), sodium-glucose cotransporter 2 (SGLT2) inhibitors, and anticoagulant therapy.

### 2.3 Outcome definition and assessment of post-discharge medication adherence

The primary outcome was unplanned rehospitalization due to worsening HF within 30 days after discharge. HF readmission was defined as hospitalization after discharge for worsening dyspnea, pulmonary congestion, peripheral edema, rapid weight gain, elevated natriuretic peptide levels, or other manifestations requiring intravenous diuretics, vasoactive agents, or other intensified anti-HF treatment (12). Admissions for non-HF causes were not counted as primary outcome events.

Patients who died within 30 days after discharge without HF readmission were not counted as primary outcome events and were recorded separately as competing events. A sensitivity analysis excluding these patients was used to assess their influence on the primary analysis. Outcome information was obtained from the hospital electronic medical record system, regional medical records, and telephone verification.

Early post-discharge medication adherence was assessed at 7 ± 2 days after discharge during an outpatient visit or by telephone follow-up using the Chinese version of the Adherence to Refills and Medications Scale (ARMS-C). If a patient was readmitted for HF before the scheduled follow-up, adherence from discharge through the day before readmission was assessed early during the rehospitalization. The ARMS-C is a 10-item self-report scale with a total score ranging from 10 to 40, with higher scores indicating poorer adherence (13). Based on evidence supporting use of the scale in studies of cardiovascular medication adherence (14) and the score distribution in this cohort, an ARMS-C total score >16 was prespecified as an exploratory, sample-specific threshold; this cutoff was not treated as an externally validated clinical diagnostic threshold. Five patients who died within 30 days after discharge without HF readmission did not complete the ARMS-C; therefore, the adherence analysis included 307 patients who completed the scale. Because the ARMS-C reflects a post-discharge behavioral factor, it was not included in the primary pre-discharge prediction model and was used only in exploratory sensitivity analysis.

### 2.4 Statistical analysis

Statistical analyses were conducted using the Statistical Package for the Social Sciences (SPSS), version 26.0, and R, version 4.3.0. Distributions of continuous variables were assessed using the Shapiro–Wilk test and histograms. Approximately normally distributed variables are presented as mean ± standard deviation and were compared between groups using the Welch independent-samples *t* test. Non-normally distributed variables are presented as median [interquartile range] and were compared using the Mann–Whitney *U* test. Categorical variables are presented as counts and percentages and were compared using the Pearson *χ*^2^ test; Fisher's exact test was used when expected frequencies were insufficient. All tests were two-sided, and *P* < 0.05 was considered statistically significant.

Patients were categorized into readmission and no-readmission groups according to whether HF readmission occurred within 30 days after discharge.

Univariable logistic regression was used to screen variables associated with 30-day HF readmission. Because NT-proBNP had a skewed distribution, it was natural-log transformed (ln) for regression analysis. Continuous variables entered the models in clinically interpretable units: per 10-year increase in age, per 10-beats/min increase in heart rate, per 10-g/L decrease in hemoglobin, per 20-μmol/L increase in serum creatinine, per 2-mmol/L increase in blood urea nitrogen, per 5-g/L decrease in albumin, per 10-mg/L increase in hs-CRP, per 10-mL/(min·1.73 m^2^) decrease in eGFR, per 10-mL/m^2^ increase in LAVI, per 5-unit increase in E/e′, per 10-mmHg increase in PASP, per 5-mm increase in IVC diameter. Odds ratios (ORs) with 95% CIs were reported for logistic regression analyses.

Given the 62 primary outcome events, multivariable logistic regression used a parsimonious modeling strategy that combined prespecified clinical variables with the results of the univariable analyses. The final multivariable model included age, NYHA class IV, atrial fibrillation, ln NT-proBNP, eGFR, E/e′, IVC-CI, and LUS B-line count. Variance inflation factors were used to assess multicollinearity. Restricted cubic splines were used to assess departures from linearity between continuous variables and the outcome logit. Model goodness of fit was evaluated using the Hosmer–Lemeshow test, and overall prediction error was assessed using the Brier score.

To determine whether pre-discharge IVC-CI and LUS B-line count provided incremental discrimination beyond standard clinical assessment, two nested models were constructed. The clinical model included age, NYHA class IV, atrial fibrillation, ln NT-proBNP, eGFR, and E/e′. The clinical-plus-ultrasound model added IVC-CI (per 5% decrease) and LUS B-line count (per 5-line increase) to the clinical model. Because the retrospective records did not systematically capture all components of the Efficacy of Vasopressin Antagonism in Heart Failure: Outcome Study With Tolvaptan (EVEREST) congestion score at the same pre-discharge time point, the formal EVEREST score was not reconstructed. AUCs for the two models were compared using the DeLong test, and optimism-corrected AUCs were obtained using 1,000 bootstrap resamples. Calibration was evaluated using the Hosmer–Lemeshow test, calibration intercept, and calibration slope, and overall prediction error was assessed using the Brier score; the calibration intercept and slope are reported after correction for optimism using 1,000 bootstrap resamples. Reclassification was evaluated using continuous net reclassification improvement (NRI), categorical NRI based on prespecified risk strata (<10%, 10%–20%, and ≥20%), and integrated discrimination improvement (IDI). Decision curve analysis (DCA) was used to compare clinical net benefit across threshold probabilities of 5%–50%.

To assess potential selection bias, the total number of patients with ADHFpEF discharged during the study period was reported, and key baseline characteristics and 30-day outcomes were compared between patients included in the analysis and those not included because pre-discharge IVC/LUS was not performed or images were non-analyzable. To assess confounding related to the COVID-19 calendar period and chronic lung disease, patients were divided by admission date into January 2020–December 2022 and January 2023–December 2024 periods. The primary model was additionally adjusted for calendar period, and interactions between calendar period and either IVC-CI or LUS B-line count were tested. The primary model was also repeated after excluding patients with stable COPD.

## 3 Results

### 3.1 Study screening and incidence of 30-day heart failure readmission

**Figure 1:**
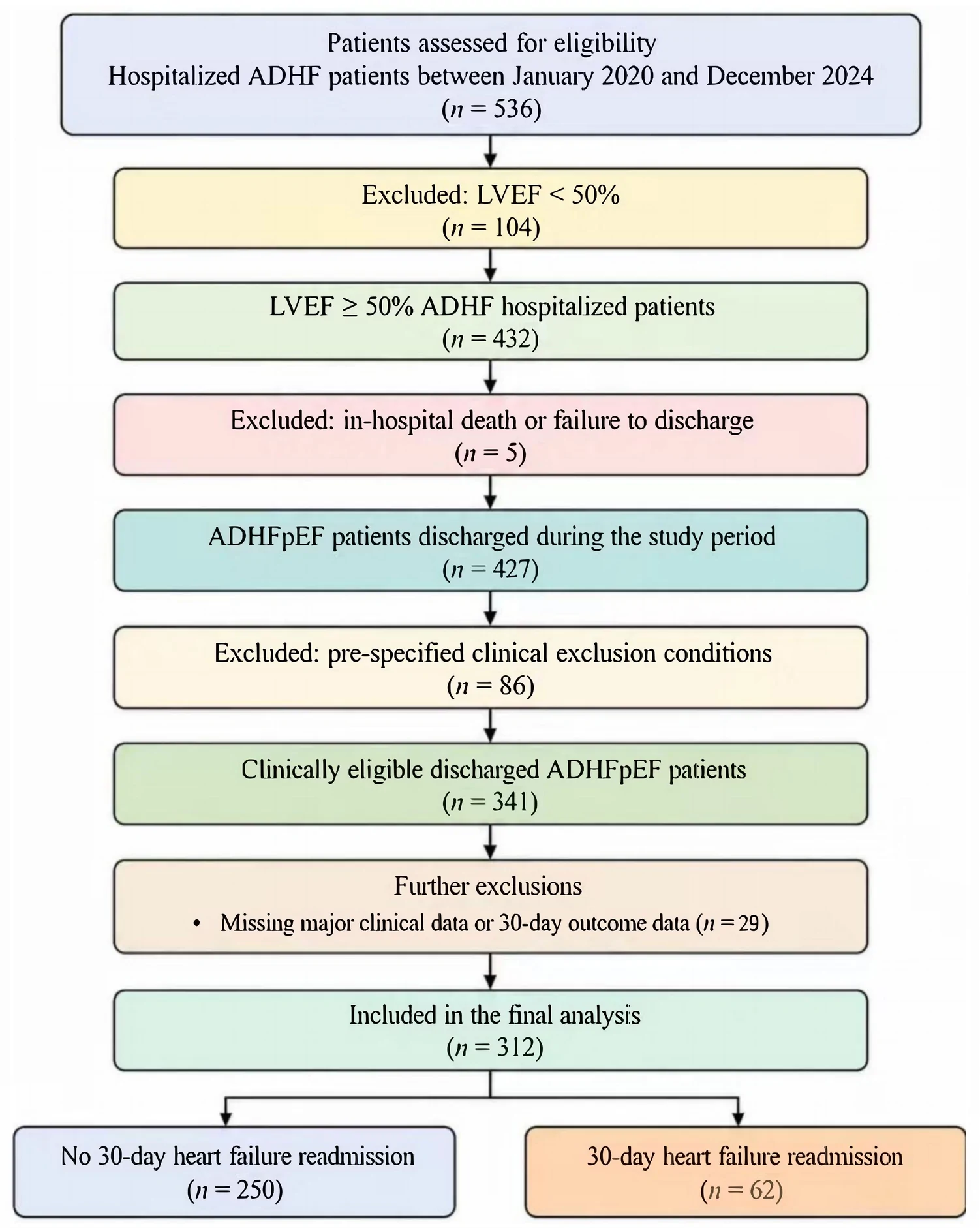
Patient screening flow diagram. *n*, number of patients; ADHF, acute decompensated heart failure; ADHFpEF, acute decompensated heart failure with preserved ejection fraction; HF, heart failure; HFpEF, heart failure with preserved ejection fraction; LVEF, left ventricular ejection fraction.

Between January 2020 and December 2024, 536 patients hospitalized with ADHF were initially screened. After 104 patients with LVEF < 50% were excluded, 432 patients hospitalized with ADHF had LVEF ≥ 50%. Of these, five died in hospital or were not discharged, leaving 427 patients with ADHFpEF discharged during the study period. A further 86 patients with prespecified clinical exclusion conditions were excluded. Among 341 clinically eligible discharged patients with ADHFpEF, 29 were excluded because key clinical data or 30-day outcome data were missing. The final analysis included 312 patients, of whom 62 experienced 30-day HF readmission and 250 did not (Figure 1). Among the 26 patients excluded because of pulmonary or systemic conditions that could affect interpretation of B-lines or volume status, acute COPD exacerbation, active pneumonia or another acute respiratory infection, COVID-19 pneumonia within 90 days or persistent noncardiogenic pulmonary parenchymal abnormalities, interstitial lung disease or pulmonary fibrosis, large pleural effusion, and confirmed cirrhosis or moderate-to-large ascites were counted preferentially according to the principal reason for exclusion (Supplementary Table S1).

### 3.2 Comparison of baseline clinical characteristics

Compared with the no-readmission group, the readmission group was older, had a longer hospital stay and a higher heart rate, and had higher proportions of NYHA class IV, atrial fibrillation, and chronic kidney disease; all differences were statistically significant (all *P* < 0.05) (Table 1).

**Table 1:** Baseline clinical characteristics according to 30-day heart failure readmission status.

Variable	Overall (*n* = 312)	No readmission (*n* = 250)	Readmission (*n* = 62)	Statistic	*P* value
Age, years	74.1 ± 8.9	73.4 ± 8.9	76.8 ± 8.2	*t*_(99.8)_ = −2.872	*P* = 0.005
Male sex, *n* (%)	131 (42.0)	102 (40.8)	29 (46.8)	*χ*^2^ (1) = 0.728	*P* = 0.394
Body mass index, kg/m^2^	26.2 ± 3.7	26.1 ± 3.6	26.8 ± 3.9	*t*_(88.5)_ = −1.284	*P* = 0.202
Smoking history, *n* (%)	83 (26.6)	64 (25.6)	19 (30.6)	*χ*^2^ (1) = 0.648	*P* = 0.421
NYHA functional class, *n* (%)				*χ*^2^ (2) = 9.068	*P* = 0.011
Class II	62 (19.9)	55 (22.0)	7 (11.3)		
Class III	192 (61.5)	156 (62.4)	36 (58.1)		
Class IV	58 (18.6)	39 (15.6)	19 (30.6)		
Length of stay, days	8 [6, 11]	8 [6, 10]	9 [7, 12]	*Z* = −2.452	*P* = 0.014
Systolic blood pressure, mmHg	131.1 ± 17.7	131.8 ± 17.4	128.2 ± 18.6	*t*_(89.3)_ = 1.381	*P* = 0.171
Diastolic blood pressure, mmHg	73.8 ± 10.9	74.2 ± 10.8	72.4 ± 11.3	*t*_(90.6)_ = 1.133	*P* = 0.260
Heart rate, beats/min	83.9 ± 13.9	82.7 ± 13.6	88.6 ± 14.2	*t*_(90.8)_ = −2.953	*P* = 0.004
Hypertension, *n* (%)	249 (79.8)	196 (78.4)	53 (85.5)	*χ*^2^ (1) = 1.547	*P* = 0.214
Diabetes mellitus, *n* (%)	137 (43.9)	105 (42.0)	32 (51.6)	*χ*^2^ (1) = 1.864	*P* = 0.172
Coronary artery disease, *n* (%)	127 (40.7)	97 (38.8)	30 (48.4)	*χ*^2^ (1) = 1.892	*P* = 0.169
Atrial fibrillation, *n* (%)	140 (44.9)	102 (40.8)	38 (61.3)	*χ*^2^ (1) = 8.432	*P* = 0.004
Chronic kidney disease, *n* (%)	100 (32.1)	70 (28.0)	30 (48.4)	*χ*^2^ (1) = 9.481	*P* = 0.002
Chronic obstructive pulmonary disease, *n* (%)	39 (12.5)	29 (11.6)	10 (16.1)	*χ*^2^ (1) = 0.932	*P* = 0.334
History of stroke, *n* (%)	42 (13.5)	31 (12.4)	11 (17.7)	*χ*^2^ (1) = 1.217	*P* = 0.270

*n*, number of patients; %, percentage. Approximately normally distributed continuous variables are presented as mean ± standard deviation, non-normally distributed continuous variables as median [interquartile range], and categorical variables as *n* (%). *t*(df), Welch independent-samples *t*-test statistic (df, degrees of freedom); *χ*^2^(df), Pearson chi-square test statistic (df, degrees of freedom); *Z*, standardized Mann–Whitney *U* test statistic. *P* values are from two-sided between-group comparisons, and *P* < 0.05 was considered statistically significant. NYHA, New York Heart Association.

### 3.3 Comparison of laboratory and echocardiographic measures

Compared with the no-readmission group, the readmission group had lower hemoglobin, albumin, and eGFR levels and higher serum creatinine, blood urea nitrogen, hs-CRP, NT-proBNP, LAVI, E/e′, and PASP values; all differences were statistically significant (all *P* < 0.05) (Table 2).

**Table 2:** Laboratory and echocardiographic measures according to 30-day heart failure readmission status.

Variable	Overall (*n* = 312)	No readmission (*n* = 250)	Readmission (*n* = 62)	Statistic	*P* value
Hemoglobin, g/L	120.7 ± 16.9	121.8 ± 16.5	116.3 ± 17.8	*t*_(88.8)_ = 2.209	*P* = 0.030
Serum creatinine, μmol/L	92 [74, 119]	89 [72, 112]	105 [82, 134]	*Z* = −3.158	*P* = 0.002
Blood urea nitrogen, mmol/L	7.4 [5.9, 9.4]	7.1 [5.8, 9.0]	8.4 [6.7, 10.9]	*Z* = −3.058	*P* = 0.002
Serum sodium, mmol/L	139.0 ± 3.8	139.1 ± 3.7	138.4 ± 4.1	*t*_(87.3)_ = 1.226	*P* = 0.223
Serum potassium, mmol/L	4.13 ± 0.46	4.11 ± 0.44	4.19 ± 0.51	*t*_(84.9)_ = −1.135	*P* = 0.260
Albumin, g/L	37.3 ± 4.2	37.6 ± 4.1	36.1 ± 4.5	*t*_(87.8)_ = 2.390	*P* = 0.019
hs-CRP, mg/L	5.1 [2.3, 11.4]	4.8 [2.1, 10.6]	6.9 [3.0, 14.8]	*Z* = −2.044	*P* = 0.041
NT-proBNP, pg/mL	2,140 [1,150, 4,050]	1,820 [1,030, 3,420]	3,650 [1,980, 6,120]	*Z* = −5.694	*P* < 0.001
eGFR, mL/(min·1.73 m^2^)	64 [49, 77]	66 [52, 78]	55 [41, 70]	*Z* = −3.390	*P* < 0.001
LVEF, %	58.5 ± 4.9	58.6 ± 4.8	57.9 ± 5.1	*t*_(89.7)_ = 0.979	*P* = 0.330
LAVI, mL/m^2^	39.3 ± 9.5	38.4 ± 9.1	42.8 ± 10.3	*t*_(86.1)_ = −3.079	*P* = 0.003
Interventricular septal thickness, mm	11.8 ± 1.9	11.7 ± 1.9	12.0 ± 2.0	*t*_(90.3)_ = −1.068	*P* = 0.289
E/e′	14.2 ± 3.8	13.7 ± 3.6	16.0 ± 4.1	*t*_(85.8)_ = −4.047	*P* < 0.001
PASP, mmHg	39.9 ± 10.2	38.9 ± 9.7	44.1 ± 11.2	*t*_(85.1)_ = −3.357	*P* = 0.001

*n*, number of patients. Approximately normally distributed continuous variables are presented as mean ± standard deviation and non-normally distributed continuous variables as median [interquartile range]. *t*(df), Welch independent-samples *t*-test statistic (df, degrees of freedom); *Z*, standardized Mann–Whitney *U* test statistic. *P* values are from two-sided between-group comparisons, and *P* < 0.05 was considered statistically significant. hs-CRP, high-sensitivity C-reactive protein; NT-proBNP, N-terminal pro-B-type natriuretic peptide; eGFR, estimated glomerular filtration rate; LVEF, left ventricular ejection fraction; LAVI, left atrial volume index; E/e′, ratio of early mitral inflow velocity to early diastolic mitral annular velocity; PASP, pulmonary artery systolic pressure.

### 3.4 Comparison of discharge medications

The proportion of patients receiving anticoagulant therapy was higher in the readmission group than in the no-readmission group, and the difference was statistically significant (*P* < 0.05) (Table 3).

**Table 3:** Discharge medications according to 30-day heart failure readmission status.

Variable	Overall (*n* = 312)	No readmission (*n* = 250)	Readmission (*n* = 62)	Statistic	*P* value
Loop diuretic, *n* (%)	278 (89.1)	220 (88.0)	58 (93.5)	*χ*^2^ (1) = 1.575	*P* = 0.209
MRA, *n* (%)	169 (54.2)	133 (53.2)	36 (58.1)	*χ*^2^ (1) = 0.474	*P* = 0.491
β-blocker, *n* (%)	189 (60.6)	154 (61.6)	35 (56.5)	*χ*^2^ (1) = 0.551	*P* = 0.458
ACEI/ARB, *n* (%)	129 (41.3)	104 (41.6)	25 (40.3)	*χ*^2^ (1) = 0.033	*P* = 0.855
ARNI, *n* (%)	51 (16.3)	42 (16.8)	9 (14.5)	*χ*^2^ (1) = 0.190	*P* = 0.663
SGLT2 inhibitor, *n* (%)	102 (32.7)	84 (33.6)	18 (29.0)	*χ*^2^ (1) = 0.471	*P* = 0.493
Anticoagulant therapy, *n* (%)	129 (41.3)	94 (37.6)	35 (56.5)	*χ*^2^ (1) = 7.280	*P* = 0.007

*n*, number of patients; %, percentage. Approximately normally distributed continuous variables are presented as mean ± standard deviation, non-normally distributed continuous variables as median [interquartile range], and categorical variables as *n* (%). *t*(df), Welch independent-samples *t*-test statistic (df, degrees of freedom); *χ*^2^(df), Pearson chi-square test statistic (df, degrees of freedom); *Z*, standardized Mann–Whitney *U* test statistic. *P* values are from two-sided between-group comparisons, and *P* < 0.05 was considered statistically significant. MRA, mineralocorticoid receptor antagonist; ACEI, angiotensin-converting enzyme inhibitor; ARB, angiotensin II receptor blocker; ARNI, angiotensin receptor-neprilysin inhibitor; SGLT2, sodium-glucose cotransporter 2.

### 3.5 Logistic regression analysis of factors associated with 30-day heart failure readmission

Univariable logistic regression showed that age, length of stay, NYHA class IV, heart rate, atrial fibrillation, chronic kidney disease, lower hemoglobin, higher serum creatinine, higher blood urea nitrogen, lower albumin, higher hs-CRP, higher ln NT-proBNP, lower eGFR, higher LAVI, higher E/e′, higher PASP, anticoagulant therapy, were associated with a higher likelihood of 30-day HF readmission (all *P* < 0.05) (Table 4). In the multivariable model, higher ln NT-proBNP, lower IVC-CI, and higher LUS B-line count were independently associated with 30-day HF readmission, whereas age, NYHA class IV, atrial fibrillation, eGFR, and E/e′ did not reach statistical significance after adjustment (Table 5).

**Table 4:** Univariable logistic regression analysis of factors associated with 30-day heart failure readmission.

Variable	OR (95% CI)	Wald *χ*^2^	*P* value
Age, per 10-year increase	1.35 (1.06–1.72)	5.91	*P* = 0.015
Length of stay, per 1-day increase	1.10 (1.02–1.19)	5.87	*P* = 0.015
NYHA class IV vs. classes II–III	2.39 (1.26–4.53)	7.12	*P* = 0.008
Heart rate, per 10-beats/min increase	1.32 (1.08–1.61)	7.43	*P* = 0.006
Atrial fibrillation	2.30 (1.30–4.06)	8.22	*P* = 0.004
Chronic kidney disease	2.41 (1.36–4.26)	9.12	*P* = 0.003
Hemoglobin, per 10-g/L decrease	1.20 (1.03–1.41)	5.18	*P* = 0.023
Serum creatinine, per 20-μmol/L increase	1.23 (1.07–1.41)	8.65	*P* = 0.003
Blood urea nitrogen, per 2-mmol/L increase	1.25 (1.08–1.44)	9.25	*P* = 0.002
Albumin, per 5-g/L decrease	1.45 (1.07–1.98)	5.60	*P* = 0.018
hs-CRP, per 10-mg/L increase	1.16 (1.01–1.34)	4.23	*P* = 0.040
ln NT-proBNP, per 1-unit increase	2.18 (1.55–3.06)	20.17	*P* < 0.001
eGFR, per 10-mL/(min·1.73 m^2^) decrease	1.31 (1.11–1.55)	10.05	*P* = 0.002
LAVI, per 10-mL/m^2^ increase	1.52 (1.12–2.06)	7.25	*P* = 0.007
E/e′, per 5-unit increase	1.88 (1.30–2.72)	11.24	*P* < 0.001
PASP, per 10-mmHg increase	1.56 (1.17–2.08)	9.18	*P* = 0.002
Anticoagulant therapy	2.15 (1.22–3.78)	7.04	*P* = 0.008

OR, odds ratio; CI, confidence interval; Wald *χ*^2^, Wald test statistic for the regression coefficient in logistic regression. *P* values are two-sided Wald test results for the corresponding regression coefficient, and *P* < 0.05 was considered statistically significant. NYHA, New York Heart Association; hs-CRP, high-sensitivity C-reactive protein; NT-proBNP, N-terminal pro-B-type natriuretic peptide; ln, natural logarithm; eGFR, estimated glomerular filtration rate; LAVI, left atrial volume index; E/e′, ratio of early mitral inflow velocity to early diastolic mitral annular velocity; PASP, pulmonary artery systolic pressure.

**Table 5:** Multivariable logistic regression analysis of factors associated with 30-day heart failure readmission.

Variable	Adjusted OR (95% CI)	Wald *χ*^2^	*P* value
Age, per 10-year increase	1.12 (0.80–1.57)	0.43	*P* = 0.510
NYHA class IV vs. classes II–III	1.34 (0.63–2.86)	0.58	*P* = 0.448
Atrial fibrillation	1.30 (0.65–2.61)	0.55	*P* = 0.459
ln NT-proBNP, per 1-unit increase	1.58 (1.04–2.39)	4.64	*P* = 0.031
eGFR, per 10-mL/(min·1.73 m^2^) decrease	1.13 (0.91–1.41)	1.20	*P* = 0.274
E/e′, per 5-unit increase	1.18 (0.76–1.83)	0.55	*P* = 0.460

Adjusted OR, odds ratio from multivariable logistic regression; CI, confidence interval; Wald *χ*^2^, Wald test statistic for the regression coefficient in logistic regression. *P* values are two-sided Wald test results for the corresponding regression coefficient, and *P* < 0.05 was considered statistically significant. NYHA, New York Heart Association; NT-proBNP, N-terminal pro-B-type natriuretic peptide; ln, natural logarithm; eGFR, estimated glomerular filtration rate; E/e′, ratio of early mitral inflow velocity to early diastolic mitral annular velocity.

Restricted cubic spline tests did not indicate nonlinear relationships between continuous variables in the final model and the outcome logit: age *χ*^2^(2) = 1.09, *P* = 0.580; ln NT-proBNP *χ*^2^(2) = 1.18, *P* = 0.554; eGFR *χ*^2^(2) = 1.65, *P* = 0.438; E/e′ *χ*^2^(2) = 1.21, *P* = 0.546; IVC-CI *χ*^2^(2) = 1.42, *P* = 0.492; and LUS B-line count *χ*^2^(2) = 1.76, *P* = 0.415.

Variance inflation factors in the multivariable model ranged from 1.03 to 1.84. The Hosmer–Lemeshow goodness-of-fit test yielded *χ*^2^(8) = 5.32, *P* = 0.723, and the Brier score was 0.126. After exclusion of the five patients who died within 30 days after discharge without HF readmission, sensitivity analyses remained consistent with the primary analysis: each 5-line increase in LUS B-line count was also associated with a higher likelihood (Wald *χ*^2^ = 12.22; adjusted OR, 1.45; 95% CI, 1.18–1.79; *P* < 0.001). After exclusion of these five patients, the combined model had an AUC of 0.869 (95% CI, 0.822–0.916) and a bootstrap-corrected AUC of 0.852.

### 3.6 Incremental discrimination beyond the clinical model

Using the available pre-discharge clinical variables, the clinical model had an AUC of 0.766 (95% CI, 0.697–0.834), a bootstrap-corrected AUC of 0.748, and a Brier score of 0.149. After IVC-CI and LUS B-line count were added to the clinical model, the AUC increased to 0.889 (95% CI, 0.844–0.934), the bootstrap-corrected AUC was 0.866, and the Brier score decreased to 0.126. The DeLong test showed an AUC increment of 0.123 for the clinical-plus-ultrasound model vs. the clinical model (95% CI, 0.064–0.182; *Z* = 4.09; *P* < 0.001). The calibration intercept was 0.00 for both models, the calibration slopes were 0.91 and 0.94, respectively, and the Hosmer–Lemeshow tests did not indicate obvious lack of fit. Continuous NRI, categorical NRI, and IDI all indicated improved reclassification.

### 3.7 Selection bias and COVID-19–related sensitivity analyses

Event rates did not differ significantly between the two COVID-19-related calendar periods.

### 3.8 Post-discharge medication adherence and exploratory sensitivity analysis

A total of 307 patients completed the ARMS-C, corresponding to a completion rate of 98.4%. The readmission group had a higher ARMS-C total score than the no-readmission group, indicating poorer medication adherence. The proportion of nonadherence defined by the prespecified exploratory threshold of ARMS-C > 16 was also higher in the readmission group than in the no-readmission group, and both differences were statistically significant (both *P* < 0.05).

## 4 Discussion

The principal findings of this study were as follows. Among 312 patients with ADHFpEF, 62 (19.9%) were readmitted for HF within 30 days after discharge.

The early post-discharge period after hospitalization for acute HF is a vulnerable phase in which recurrent decompensation events cluster, and residual congestion, insufficient treatment adjustment, and inadequate continuity of follow-up may all increase the short-term event burden. Metra et al. emphasized that thorough assessment of residual or recurrent congestion before discharge and continued optimization of treatment during early follow-up are important for reducing post-discharge risk (15). Garcia-Gutierrez et al. found that residual dyspnea, frailty, and worsening quality of life were associated with readmission within 1 month, suggesting that assessment of discharge readiness should extend beyond whether symptoms have temporarily improved (16).

In the multivariable model, ln NT-proBNP remained associated with 30-day HF readmission, whereas eGFR, E/e′, and atrial fibrillation did not reach statistical significance after joint adjustment. This finding may reflect overlap in information among variables, limitations in sample size and event count, and heterogeneity of the HFpEF phenotype; these measures should nevertheless continue to be interpreted in the context of established evidence and the clinical setting. The 2023 focused update from the European Society of Cardiology (ESC) emphasized that HFpEF management should encompass volume control, management of comorbidities, and evidence-based pharmacotherapy (17). Solomon et al. showed that dapagliflozin reduced the risk of worsening HF or cardiovascular death in patients with mildly reduced or preserved ejection fraction (18).

The post-discharge medication adherence analysis showed higher ARMS-C scores and a higher proportion of nonadherence in the readmission group. Deek and Massouh found that anxiety, follow-up status, quality of life, and functional class were associated with medication adherence in patients with HF (19). Zhang et al. reported that mobile health interventions can improve medication adherence and reduce readmission in patients with HF (20). Because the ARMS-C was assessed after discharge, it was used only in exploratory sensitivity analysis and not as a baseline variable in the pre-discharge prediction model.

Using machine learning, Kyodo et al. identified HFpEF phenogroups with distinct clinical characteristics and prognoses, further supporting multidimensional phenotype integration in HFpEF risk assessment (21).

This study has several limitations. First, it was a single-center retrospective cohort. Although consecutive screening, prespecified eligibility criteria, a study flow diagram, and comparison of included and non-included patients were used, selection bias and residual confounding may remain. Fourth, the ARMS-C was assessed early after discharge, and ARMS-C > 16 was a sample-specific exploratory threshold that has not been externally validated. Patients who died within 30 days without HF readmission were recorded only as competing events, and a formal competing-risk model was not used. The retrospective records also did not fully capture all components of the EVEREST congestion score.

## 5 Conclusions

In patients with ADHFpEF, 30-day heart failure readmission remained clinically relevant and was associated with selected clinical and laboratory characteristics, particularly higher NT-proBNP levels. Poor post-discharge medication adherence was also associated with an increased risk of early readmission in exploratory analyses. These findings highlight the importance of comprehensive pre-discharge risk assessment, optimization of evidence-based therapy, and close follow-up with adherence support after discharge. Further multicenter prospective studies are needed to validate these findings and establish a robust risk-stratification approach for early readmission.

## Data Availability

The original contributions presented in the study are included in the article/Supplementary Material, further inquiries can be directed to the corresponding author.
