# Supplementary material for: Clinical factors associated with 30-day heart failure readmission in patients with acute decompensated heart failure with preserved ejection fraction
Source: Front Cardiovasc Med. 2026 Aug 19;13:1891685. doi: 10.3389/fcvm.2026.1891685 (PMC13533898; doi:10.3389/fcvm.2026.1891685)
Supplement: Supplementary file 1 [file Table1.docx]

**Supplementary Materials**

**Supplementary Table 1. Distribution of exclusion reasons among 26 patients with pulmonary or systemic conditions that could interfere with ultrasound interpretation**

| Reason for exclusion | n | Description |
| --- | --- | --- |
| Acute exacerbation of chronic obstructive pulmonary disease | 5 | Airflow limitation and pulmonary hyperinflation during acute exacerbation may affect interpretation of B-lines and volume status |
| Active pneumonia or other acute respiratory infection | 7 | Active infection can produce noncardiogenic B-lines and reduce the specificity of B-lines for pulmonary congestion. |
| COVID-19 pneumonia within 90 days or persistent noncardiogenic pulmonary parenchymal abnormalities | 4 | Recent or residual COVID-19-related pulmonary parenchymal changes can produce noncardiogenic B-lines |
| Interstitial lung disease or pulmonary fibrosis | 4 | Diffuse interstitial abnormalities can mimic or coexist with B-lines caused by pulmonary congestion. |
| Large pleural effusion | 2 | Pleural fluid can affect the local lung ultrasound acoustic window and B-line interpretation |
| Confirmed cirrhosis or moderate-to-large ascites | 4 | Ascites and portal hypertension-related abnormal fluid distribution may affect IVC collapsibility and volume status assessment |
| Total | 26 | Counted preferentially according to the main reason for exclusion; the total was 26 patients |

**Note:** n = number of patients. Patients were counted according to the principal exclusion reason. When more than one interfering condition was present, only the condition judged most likely to affect B-line or volume-status interpretation was counted. COPD = chronic obstructive pulmonary disease; COVID-19 = coronavirus disease 2019; IVC = inferior vena cava; LUS = lung ultrasound.

**Supplementary Table 2. Comparison of key characteristics between included patients and patients excluded because pre-discharge inferior vena cava ultrasonography or lung ultrasound was not performed, or images were not analyzable**

| Variable | Included patients (*n* = 312) | Ultrasound not performed or images not analyzable (n = 18) | Statistic or effect estimate | *P* value |
| --- | --- | --- | --- | --- |
| Age, years | 74.1 ± 8.9 | 75.0 ± 9.2 | *t* = −0.40 | *P* = 0.691 |
| Male sex, *n* (%) | 131 (42.0) | 8 (44.4) | *χ²* = 0.04 | *P* = 0.837 |
| Body mass index, kg/m² | 26.2 ± 3.7 | 26.5 ± 3.9 | *t* = −0.32 | *P* = 0.754 |
| NYHA class IV, *n* (%) | 58 (18.6) | 4 (22.2) | OR = 1.25 | *P* = 0.756 |
| Atrial fibrillation, *n* (%) | 140 (44.9) | 9 (50.0) | *χ²* = 0.18 | *P* = 0.671 |
| Chronic kidney disease, *n* (%) | 100 (32.1) | 7 (38.9) | *χ²* = 0.36 | *P* = 0.547 |
| NT-proBNP, pg/mL | 2140 [1150, 4050] | 2320 [1240, 4380] | *Z* = −0.54 | *P* = 0.590 |
| eGFR, mL/(min·1.73 m²) | 64 [49, 77] | 61 [46, 76] | *Z* = −0.50 | *P* = 0.620 |
| LVEF, % | 58.5 ± 4.9 | 58.0 ± 5.3 | *t* = 0.39 | *P* = 0.700 |
| 30-day HF readmission, *n* (%) | 62 (19.9) | 4 (22.2) | OR = 1.15 | *P* = 0.765 |

**Note:** n = number of patients; % = percentage. Continuous variables are expressed as mean ± standard deviation or median [interquartile range]; categorical variables are expressed as n (%). *t* = Welch independent-samples *t*-test statistic; *χ²* = Pearson chi-square test statistic; *Z* = standardized Mann–Whitney *U* test statistic; OR = odds ratio. For the New York Heart Association class IV and 30-day heart-failure readmission rows, the OR compares the ultrasound-not-performed/non-analyzable group with the included group and the two-sided *P* value was obtained using Fisher’s exact test; the OR is an effect estimate rather than a test statistic. *P* < 0.05 was considered statistically significant. NYHA = New York Heart Association; NT-proBNP = N-terminal pro-B-type natriuretic peptide; eGFR = estimated glomerular filtration rate; LVEF = left ventricular ejection fraction; HF = heart failure.

**Supplementary Table 3. Sensitivity analyses for coronavirus disease 2019–related calendar periods and stable chronic obstructive pulmonary disease**

| Analysis | Estimate | Statistic | *P* value |
| --- | --- | --- | --- |
| Event rate by period | 2020–2022: 36/178 (20.2%); 2023–2024: 26/134 (19.4%) | *χ²* = 0.03 | *P* = 0.857 |
| Main model + calendar period | IVC-CI per 5% decrease: OR 1.34 (1.09–1.64) | Wald *χ²* = 7.89 | *P* = 0.005 |
| Main model + calendar period | LUS B-lines per 5-line increase: OR 1.46 (1.19–1.80) | Wald *χ²* = 12.85 | *P* < 0.001 |
| Main model + calendar period | Calendar period (2020–2022 vs 2023–2024): OR 1.03 (0.56–1.90) | Wald *χ²* = 0.01 | *P* = 0.930 |
| 2020–2022 stratified analysis | IVC-CI: OR 1.32 (1.04–1.68); LUS B-lines: OR 1.46 (1.13–1.88) | Wald *χ²* = 5.15; 8.49 | *P* = 0.024; *P* = 0.004 |
| 2023–2024 stratified analysis | IVC-CI: OR 1.36 (1.03–1.80); LUS B-lines: OR 1.49 (1.13–1.96) | Wald *χ²* = 4.66; 8.06 | *P* = 0.030; *P* = 0.005 |
| Interaction terms | IVC-CI × period; LUS B-lines × period | Interaction-term Wald *χ²* = 0.02; 0.04 | *P* = 0.884; *P* = 0.842 |
| Excluding stable COPD | *n* = 273, events = 52; IVC-CI: OR 1.32 (1.07–1.63) | Wald *χ²* = 6.69 | *P* = 0.009 |
| Excluding stable COPD | *n* = 273, events = 52; LUS B-lines: OR 1.44 (1.16–1.79) | Wald *χ²* = 10.86 | *P* = 0.001 |

**Note:** The estimate column reports event rates, adjusted odds ratios (ORs) with 95% confidence intervals (CIs), or period-stratified adjusted ORs. Wald *χ²* = Wald test statistic from logistic regression; *χ²* = Pearson chi-square test statistic. The main model included age, New York Heart Association class IV, atrial fibrillation, natural-log-transformed N-terminal pro-B-type natriuretic peptide, estimated glomerular filtration rate, the ratio of early mitral inflow velocity to early diastolic mitral annular velocity (E/e′), inferior vena cava collapsibility index, and lung-ultrasound B-line count; calendar period was added where indicated. Stratified models used the same covariates within each calendar period. Interaction terms tested whether calendar period modified the association of either ultrasound measure with readmission. *P* values are two-sided, and *P* < 0.05 was considered statistically significant. COPD = chronic obstructive pulmonary disease; COVID-19 = coronavirus disease 2019; IVC-CI = inferior vena cava collapsibility index; LUS = lung ultrasound; OR = odds ratio; CI = confidence interval.

**Supplementary Table 4. Chinese version of the Adherence to Refills and Medications Scale (ARMS-C) assessment and exploratory sensitivity analysis**

| Indicator | Overall/model | No readmission | Readmission | Statistic | *P* value |
| --- | --- | --- | --- | --- | --- |
| ARMS-C completion rate | 307/312 (98.4%) | 245/250 (98.0%) | 62/62 (100%) | Completion rate = 98.4% | Not applicable |
| ARMS-C total score | 15 [13, 18] | 15 [13, 18] | 17 [14, 20] | *Z* = −2.58 | *P* = 0.010 |
| Nonadherence defined by ARMS-C > 16 | 103/307 (33.6%) | 75/245 (30.6%) | 28/62 (45.2%) | *χ²* = 4.70 | *P* = 0.030 |
| Exploratory model: IVC-CI per 5% decrease | Adjusted OR 1.30 (1.06–1.60) | Not applicable | Not applicable | Wald *χ²* = 6.24 | *P* = 0.012 |
| Exploratory model: LUS B-lines per 5-line increase | Adjusted OR 1.42 (1.15–1.76) | Not applicable | Not applicable | Wald *χ²* = 10.43 | *P* = 0.001 |
| Exploratory model: ARMS-C nonadherence | Adjusted OR 1.78 (1.01–3.15) | Not applicable | Not applicable | Wald *χ²* = 3.95 | *P* = 0.047 |

**Note:** n = number of patients; % = percentage. Continuous variables are expressed as median [interquartile range]; categorical variables are expressed as n (%). ARMS-C = Chinese version of the Adherence to Refills and Medications Scale; OR = odds ratio; CI = confidence interval; Wald *χ²* = Wald test statistic from logistic regression; *Z* = standardized Mann–Whitney *U* test statistic; *χ²* = Pearson chi-square test statistic. Adjusted ORs are reported with 95% CIs. The exploratory multivariable model added ARMS-C nonadherence to the main multivariable model. Nonadherence was defined as ARMS-C total score > 16 solely as a prespecified, sample-specific exploratory threshold and not as an externally validated clinical diagnostic cutoff. *P* values are two-sided, and *P* < 0.05 was considered statistically significant. IVC-CI = inferior vena cava collapsibility index; LUS = lung ultrasound.
